# Unmasking the Archnemesis: Primary Hyperparathyroidism Presenting As Brown Tumors of the Jaws

**DOI:** 10.7759/cureus.84427

**Published:** 2025-05-19

**Authors:** Sakshi Batra, Adit Srivastava, Saumya Shukla

**Affiliations:** 1 Oral Medicine and Radiology, Banaras Hindu University, Varanasi, IND; 2 Oral Medicine and Radiology, Faculty of Dental Sciences, Institute of Medical Sciences, Banaras Hindu University, Varanasi, IND; 3 Oral Medicine and Radiology, Faculty of Dental Sciences, Banaras Hindu University, Varanasi, IND

**Keywords:** brown tumors, osteitis fibrosa cystica, parathyroid adenoma, parathyroidectomy, primary hyperparathyroidism

## Abstract

Brown tumor (BT), particularly of the jaws, is a rare and late manifestation of primary hyperparathyroidism (PHPT), a disease most commonly reported in middle-aged women. It is usually attributed to adenoma (single or multiple), hyperplasia of the parathyroid gland, and rarely carcinoma. A generalized loss of lamina dura, although a late manifestation, is most commonly attributed to PHPT. The routine use of orthopantomograms (OPG) in dentistry makes the dentist one of the frontrunners in diagnosing this disease.

Here we report the case of a 42-year-old female who presented to our clinic with the complaint of swelling in the right back region of the lower jaw and loosening of teeth. OPG and cone-beam computed tomography revealed a generalized loss of lamina dura and three well-defined radiolucent regions: two in the mandible and one in the maxilla. The provisional diagnosis was giant cell lesion of hyperparathyroidism, which was further confirmed by biochemical analysis and sestamibi scan. The patient underwent left parathyroidectomy, and clinical and radiographic follow-up was done at 6 months and 12 months after surgery.

Our patient had a single adenoma on her left parathyroid gland, leading to primary hyperparathyroidism, which only presented with oral symptoms. This case highlights the importance of dentists in diagnosing such endocrinopathies that may present with mere oral manifestations.

## Introduction

Primary hyperparathyroidism (PHPT) is a significant endocrine disorder characterized by the overproduction of parathyroid hormone (PTH), primarily affecting women in their 40s [1,2]. The predominant cause of PHPT is a single adenoma, accounting for 75%-85% of cases, followed by hyperplasia of the parathyroid gland (10%-20%), multiple adenomas (4%-5%), and carcinoma (1%) [1]. The diagnosis of PHPT is established through the detection of hypercalcemia and elevated PTH levels [3].

Genetic syndromes causing PHPT include multiple endocrine neoplasia (MEN type 1 or 2A), familial hypocalciuric hypercalcemia (FHH), and hyperparathyroidism jaw-tumor syndrome [1,2]. The disease was described by Fuller Albright and others as a “disease of stones, bones, and groans” [3]. Numerous organs are impacted by PHPT, such as the central nervous system, kidneys, soft tissues, bone, and the gastrointestinal tract. The odontogenic tissue is impacted as well, oftentimes after the disease has reached an advanced stage. The most frequent observations on radiographs include ground glass appearance of the jaw bones and complete or partial loss of lamina dura (LD) [4].

In the 1970s, mild hypercalcemia became easily detectable, so the routine use of serum calcium, as part of a biochemical screening profile, became a common practice, and most people are asymptomatic at the time of diagnosis [1]. However, in developing and underdeveloped countries, this is not the norm, and the disease may advance undetected.

The radiographic manifestation of PHPT is linked to certain skeletal anomalies, such as brown tumors (BT), distal tapering of the phalanges and clavicles, subperiosteal bone resorption, and a "salt-and-pepper" appearance of the skull. These abnormalities are collectively referred to as "osteitis fibrosa cystica" [3]. Despite being called tumors, BTs are essentially non-neoplastic giant cell lesions that take on their distinctive brown color due to excessive osteoclastic activity, bone resorption, fibrous tissue replacement, and hemosiderin deposition [4].

A small subset of PHPT patients, approximately 5.9% according to a retrospective study in India, present with giant cell lesions such as BTs [4]. These lesions can manifest as palpable swellings, often associated with bone pain or pathological fractures [1,5]. Radiographically, BTs typically appear as well-defined osteolytic lesions with a soap bubble or multilocular appearance, potentially mimicking other conditions such as multiple myeloma or metastatic carcinoma [6,7].

Biochemical analysis plays a crucial role in distinguishing BTs from malignant lesions, as PHPT is marked by significantly elevated serum calcium and PTH levels, which are not observed in malignancies [8]. Histologically, BTs can resemble giant cell tumors (GCT), giant cell reparative granulomas (GCRG), and solid variant aneurysmal bone cysts (SV-ABC), necessitating careful differential diagnosis [9].

Surgical intervention, especially parathyroidectomy, is usually required for PHPT management and can result in regression [7]. However, bisphosphonates and calcimimetic drugs have been tried. Oral medicine and radiology specialists are often the first to identify radiographic abnormalities suggestive of PHPT. Upon detecting such abnormalities, they can recommend further clinical and biochemical evaluation to confirm the diagnosis. Elevated serum calcium and PTH levels are indicative of PHPT and can be correlated with radiographic findings to establish a definitive diagnosis. This approach enhances diagnostic accuracy and timely patient care.

## Case presentation

A 42-year-old female patient reported to the Department of Oral Medicine and Radiology with the chief complaint of swelling of gums on the right lower back region of the oral cavity for the past 6 months, with loosening of teeth and difficulty in chewing food. The patient had a history of trauma to the chin 14 years back, for which she was treated with splinting. She mentioned generalized body pain and lethargy. No relevant family history was present. No history of fever, pus discharge, toothache, and no other associated symptoms such as abdominal pain, renal colic, or pain in other bones were mentioned.

**Figure 1:**
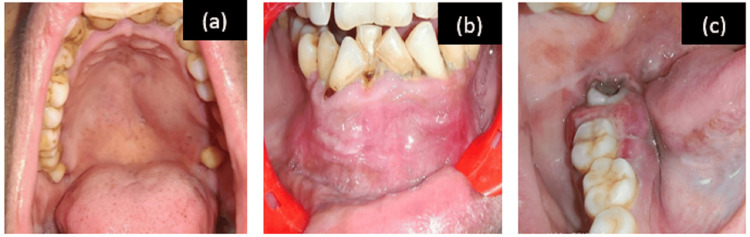
Intraoral pictures Intraoral pictures of (a) dome-shaped swelling in the left side of the palate, (b) dome-shaped swelling in the anterior mandible obliterating the labial vestibule, and (c) swelling on the right side of the posterior mandible.

On intra-oral examination, three swellings were present in the oral cavity: two swellings were dome-shaped present in the upper posterior palatal region extending from 24 to 27, not crossing the midline of the palate, and in the lower anterior region of the jaw extending from 34 to 44, measuring approximately 3x2.5 cm and 5x4.5 cm, respectively; the third swelling was well defined surrounding 47, 48 measuring approximately 3x 2.5 cm, obliterating the buccal vestibule and displacing 47 buccally and 48 more distally. All were firm to hard in consistency and mildly tender. 48 was carious, and malocclusion was present with generalized recession of the gingiva (Figure 1).

Based on these findings, a provisional diagnosis of chronic generalized periodontitis was made with a giant cell lesion of the mandibular anterior region.

**Figure 2:**
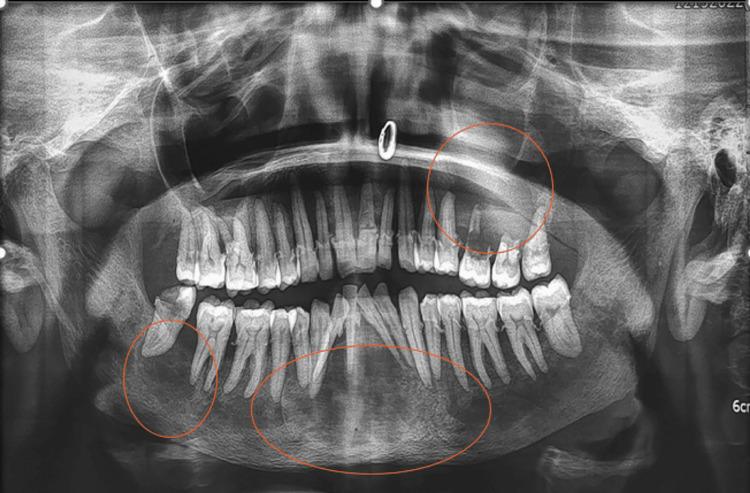
Orthopantomogram Orthopantomogram (OPG) revealing a generalised loss of lamina dura. Well-defined radiolucencies with non-corticated borders are noted in the left posterior maxillary and right posterior mandibular regions. Another well-defined, unilocular radiolucency with corticated borders showing heterogenous radiolucent to radio-opaque features is seen in the mandibular symphysis.

Routine investigations (complete hemogram and blood sugar) and an orthopantomogram (OPG) were advised. The OPG revealed a generalized loss of lamina dura and variation in bone trabeculation and density, accompanied by generalized horizontal bone loss with multiple radiolucent lesions (Figure 2).

**Figure 3:**
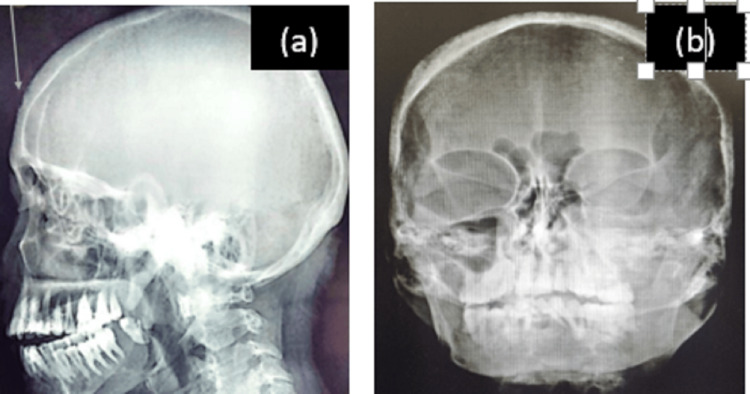
Skull radiograph (a) Lateral skull projection showing surface resorption of the skull, (b) PA skull projection showing salt and pepper appearance of the skull.

Based on this, biochemical evaluation for PTH, triiodothyronine (T3), thyroxine (T4), thyroid-stimulating hormone (TSH), Vitamin D, serum calcium, alkaline phosphatase (ALP), and phosphorus was carried out along with a skull radiograph (lateral and PA-skull view) (Figure 3).

**Figure 4:**
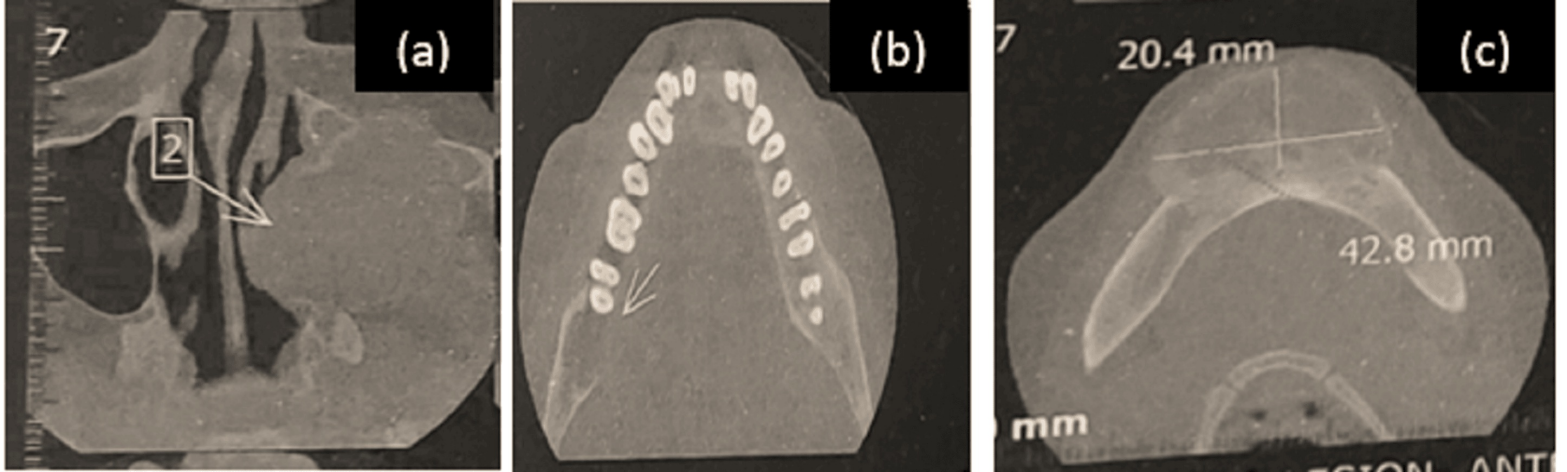
Cone beam computed tomography (CBCT) Cone beam computed tomography (CBCT) imaging showing (a) axial section revealing heterogenous radio-opaque to radiolucent expansile lesion involving the left maxillary sinus, (b) axial section revealing a radiolucency with non-corticated borders in the right posterior mandibular region, with a breach in the lingual cortex, and (c) axial section revealing a heterogenous radiopaque to radiolucent expansile lesion in the mandibular symphysis.

A cone beam computed tomography (CBCT) scan performed prior to the patient's visit to us was reviewed for evaluation and the extent of the lesion (Figure 4).

Biochemical evaluation revealed increased PTH (942.7 mg/dl), serum calcium 11 mg/dl, ALP 681 IU/l, and decreased phosphorus (2.57 mg/dl) (Table 1).

**Table 1:** Lab investigations T3: triiodothyronine; T4: thyroxine; TSH: thyroid-stimulating hormone; PCV: packed cell volume; MCV: mean corpuscular volume; MCH: mean corpuscular hemoglobin; MCHC: mean corpuscular hemoglobin concentration; TLC: total leukocyte count

Parameters	Patient’s Value	Reference Range
Parathyroid Hormone	942.7 pg/ml	15-65 pg/mL
Serum calcium	11 mg/dl	8.6-10 mg/dl
Serum Alkaline Phosphatase	681 IU/L	44-147 IU/L
Phosphorous	2.57 mg/dl	3.4-4.5 mg/dl
T3	3.45 pg/mL	2.30-4.20 pg/mL
T4	1.40 ng/dL	0.89-1.76 ng/dL
TSH	1.036 µIU/mL	0.550-4.780 µIU/mL
Vitamin D	8.42 ng/mL	Optimal 50-70 ng/mL
		Insufficient 30-50 ng/mL
		Deficient <30 ng/mL
Haemogram		
Haemoglobin	12.00 g/dl	12.00-15.00 g/dl
PCV	41.80%	36.00-46.00 %
RBC Count	4.48 million/mm3	3.80-4.80 million/mm^3^
MCV	93.30 fL	83.0-101.0 fL
MCH	26.60 pg	27.00-32.00 pg
MCHC	28.70 g/dl	31.50-34.50 g/dl
TLC	9.59 thous/mm3	4.00-10.00 thous/mm^3^
Mean Platelet Volume	13.5fL	6.0-12.0 fL
Ferritin	26.39 mg/mL	13.0-150.0 mg/mL
Fasting Blood Sugar	90 mg/dL	70.00-100.00 mg/dL
Post-prandial Blood Sugar	130 mg/dL	70.00-140.00 mg/dL

The lateral skull showed surface resorption of the skull and disappearance of the outer skull table. Based on this, a diagnosis of hyperparathyroidism with jaw lesions was made, and the patient was further referred for an endocrinology opinion.

**Figure 5:**
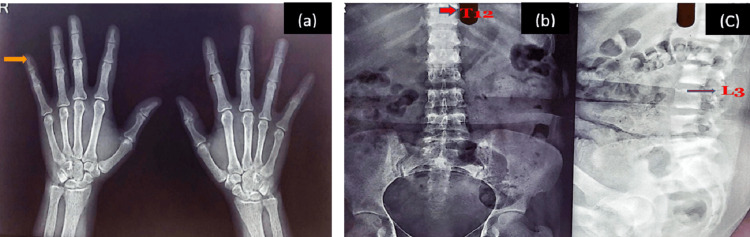
X-ray bilateral hand and an X-ray spine (thoracolumbar region) (a) Bilateral hand X-ray revealing resorption of terminal phalanges and osteopenia, (b) X-ray of the spine revealing concave appearance of T12, and (c) X-ray spine lateral view showing irregular upper and lower surface of L3.

**Figure 6:**
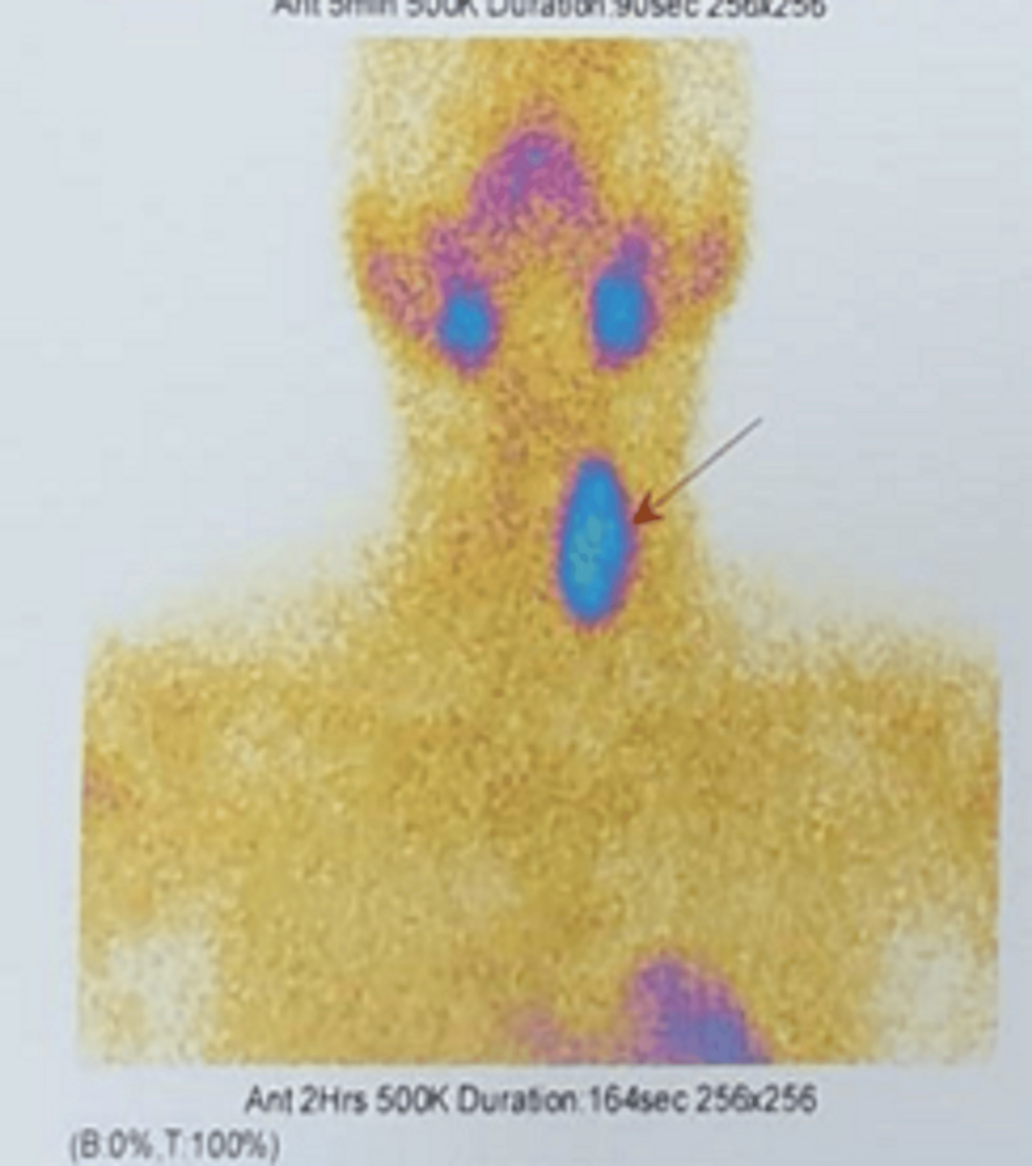
Sestamibi scan showing persisting abnormally increased focal tracer concentration in the region of enlarged left lobe of thyroid gland

Patient was evaluated in the endocrinology department and was advised for an X-ray bilateral hand and an X-ray spine (thoracolumbar region) (Figure 5). Ultrasound sonography (USG) neck and abdomen, dual-energy X-ray absorptiometry (DXA) scores, vitamin D level evaluation, and sestamibi scan (99m Tc-mibi parathyroid scan) were also carried out. X-ray of bilateral hands showed osteopenia and resorption of the terminal phalanges. X-ray spine (thoracolumbar region) showed a biconcave appearance of T12, with the upper and lower surfaces of L3 irregular. USG neck showed left parathyroid gland enlargement (1.5 x 6 cm) with presence of mass lesion arising from it of size (2.5 x 1.3 cm) with internal solid cystic areas and significant internal vascularity. USG abdomen showed the presence of mild splenomegaly. DXA scores were as follows: L1-L4 Z SCORE: -2.1, RADIUS Z SCORE: -3.9, and 25-0H Vitamin D levels were 8.42ng/ml. Sestamibi scan showed an intrathyroidal parathyroid adenoma in the enlarged left lobe of the thyroid gland (Figure 6).

**Figure 7:**
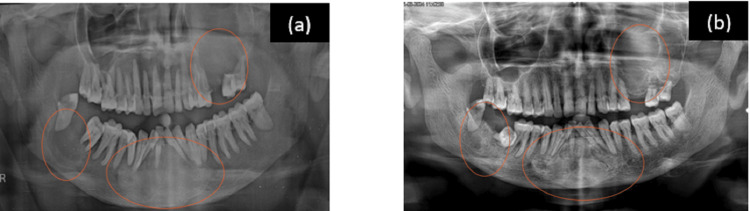
Orthopantomogram (OPG) (a) OPG taken at 6 months post-op showing increased radiopacity in the mandibular symphyseal lesion and the left maxillary region, and an increase in the size of the right posterior mandibular region, (b) all three lesions revealing an increase in radiopacity at 12 months post-op.

**Figure 8:**
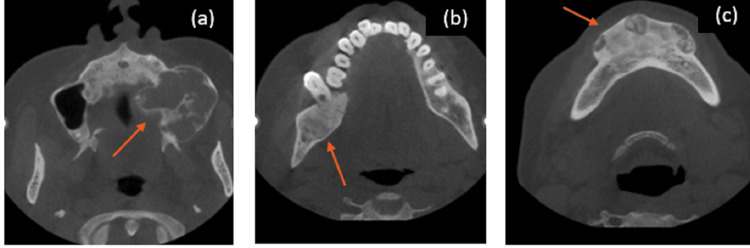
Cone beam CT (CBCT) scans at 12 months post-op CBCT scans at 12 months post-op showing (a) axial section of maxillary lesion revealing radiolucency with radiopaque flecks suggestive of osteoid formation, (b) axial section of posterior mandibular lesion showing complete opacification of the lesion along with resolution of the breach in the lingual cortex, and (c) axial section of the mandible showing opacification of the symphyseal lesion.

**Figure 9:**
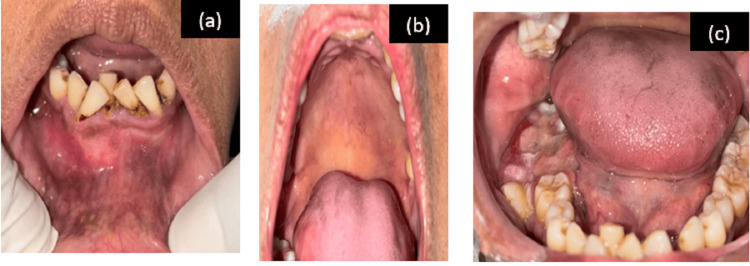
Intraoral images taken after 12 months Intraoral images taken after 12 months showing (a) regression of the swelling in the mandibular anterior region, (b) nearly completely resolved palatal swelling, and (c) no further increase in the size of the swelling in the right mandibular posterior region.

All the investigations led to the final diagnosis of left inferior parathyroid adenoma with cystic jaw lesions. She underwent left parathyroidectomy after the diagnosis of intrathyroidal parathyroid adenoma in the enlarged left lobe of the thyroid gland (as per the sestamibi scan). Six months post-op imaging showed increased radiopacity within the mandibular lesion, and tooth mobility was clinically reduced with no further enlargement of the swellings. A marked decrease in the size of the swellings, increased radiopacity of the maxillary lesion, partial formation of lamina dura, and nearly complete resolution of the mandibular lesions were seen 12 months after surgery (Figures 7-9).

This demonstrates how maxillofacial surgery would have only dealt with what was the tip of the iceberg, masking a larger underlying systemic cause.

## Discussion

The diagnosis of PHPT is established in the presence of hypercalcemia and elevated PTH levels. Radiographically, PHPT is characterized by subperiosteal bone resorption, distal tapering of the clavicles and phalanges, a “salt-and-pepper” appearance of the skull, and BTs. These features, clubbed together, are known as “osteitis fibrosa cystica” [3].

PTH plays an important role in calcium homeostasis in the following ways: (1) it stimulates osteoclasts, leading to bone resorption, (2) it augments the conversion of vitamin D to its active form, i.e. vitamin D (1,25-dihydroxycholecalciferol), and (3) it acts on the distal tubule to increase reabsorption of calcium. An imbalance in osteoclastic and osteoblastic activity results in bone resorption, fibrous replacement of the marrow, and cortical thinning, which leads to the formation of a brown tumor [4].

It is rare to discover BTs serving as the primary symptom of hyperparathyroidism; they might appear as a palpable swelling in the region of concern, which may be associated with deep pain or pathologic fracture [1,5].

The severity of PHPT was correlated with loss of LD, decreased mandibular cortical width, and a ground-glass look of the jaw bone on conventional radiography. A small increase in PTH or ALP did not change the odontogenic tissue, while a substantial rise in these parameters was associated with a higher frequency of absence of LD. Therefore, it seemed that the odontogenic symptoms and signs were an indication of PHPT's advanced stages [4]. This was evident in the case of our patient, whose PTH levels showed an alarming aberration.

In the case of a lytic lesion of the jaw bones, the most likely diagnoses would include odontogenic cysts and tumors (radicular cyst, lateral periodontal cyst, and ameloblastoma), primary bone tumors and cysts (simple bone cyst, eosinophilic granuloma, giant cell lesions, odontogenic keratocyst, myxoma, and odontogenic fibroma), infectious diseases (bone abscess, localized osteomyelitis), metabolic bone disease, hyperparathyroidism, hyperparathyroidism-jaw tumor syndrome, and metastasis from a known or an unknown primary site (lung, breast, kidney, prostate) [10]. It is quite possible to misdiagnose multiple bone lesions of brown tumor as multiple myeloma, metastatic carcinoma, lymphangiomatosis, leukemia, Langerhans' cell histiocytosis, multiple bone cysts, or multiple non-ossifying fibromas based on similar radiographic appearances [7].

Looking at the OPG, the most prominent disease that came to mind was giant cell lesion of hyperparathyroidism, although multiple odontogenic keratocysts (due to multiple radiolucent lesions and age of the patient) and ameloblastoma (due to root resorption seen associated with the maxillary lesion) were also considered. The loss of lamina dura could also be attributed to osteoporosis, which is again commonly seen in older women. Paget’s disease is another such disease that may cause bone remodeling, a generalized loss of lamina dura, and is seen in the older population. This is where biochemical analysis comes to the rescue.

Dissimilarities in biochemistry and clinical manifestations are crucial in distinguishing these entities. Along with elevated levels of PTH and ALP, the hallmark abnormalities of PHPT include hypercalcemia, hypophosphatemia, and hypercalciuria [7].

In our case, biochemical analysis revealing raised PTH and calcium levels led us to the diagnosis of PHPT, which, as found on further investigations like USG and sestamibi scans, was caused by a single adenoma on the left parathyroid gland.

In terms of histology, BTs can be mistaken for GCTs, GCRGs, and SV-ABCs. GCT is a benign tumor that is defined by an overabundance of stromal round mononuclear cells and multinuclear giant cells that resemble mature osteoclasts. GCT and BT are similar in that they are made up of a mixture of multinuclear giant cells and mononuclear cells. On the other hand, it has been shown that giant cells typically cluster in BTs, but they are generally dispersed evenly in GCT. Compared to GCRG or SV-ABC, Brown tumor exhibits a significantly more lobulated architectural growth pattern. However, in smaller biopsy samples, these lesions might not be readily differentiated [9].

Alendronate or raloxifene therapy may be explored for patients with osteoporosis caused by hyperparathyroidism. While there is currently no recognized medical treatment for PHPT, PTH and serum calcium levels may be lowered with calcimimetics [11]. Spontaneous regression of BT may be achieved by performing parathyroidectomy [7], as seen in our case. Surgical correction may be required with larger, disfiguring lesions.

## Conclusions

The mouth is the mirror of the body, often displaying signs of systemic diseases before they fully develop. This case underlines the importance of considering systemic conditions in the differential diagnosis of jaw lesions. Early recognition and referral for appropriate medical evaluation are crucial in managing such cases effectively.

Therefore, routine dental examinations serve not only to preserve oral well-being but also potentially unveil hidden diseases. Through awareness and vigilance, dentists can play a crucial role in promptly identifying and addressing such conditions, ultimately saving patients' time and, in some cases, their lives.
